# Long-Term Dietary Intake of Chia Seed Is Associated with Increased Bone Mineral Content and Improved Hepatic and Intestinal Morphology in Sprague-Dawley Rats

**DOI:** 10.3390/nu10070922

**Published:** 2018-07-19

**Authors:** Evelyn M. Montes Chañi, Sandaly O. S. Pacheco, Gustavo A. Martínez, Maykon R. Freitas, Joaquin G. Ivona, Javier A. Ivona, Winston J. Craig, Fabio J. Pacheco

**Affiliations:** 1 Center for Health Sciences Research, School of Medicine & Health Sciences, Universidad Adventista del Plata, Libertador San Martín, Entre Ríos 3103, Argentina; evymar80@gmail.com (E.M.M.C.); sandaly.oliveira@uap.edu.ar (S.O.S.P.); gustimart3@gmail.com (G.A.M.); maykonrocha.med@gmail.com (M.R.F.); joaquin.ivona@gmail.com (J.G.I.); javier.ivona@gmail.com (J.A.I.); wcraig@andrews.edu (W.J.C.); 2 Institute for Food Science and Nutrition, Universidad Adventista del Plata, Libertador San Martín, Entre Ríos 3103, Argentina; 3 Department of Public Health, Nutrition and Wellness, School of Health Professions, Andrews University, Berrien Springs, MI 49104, USA

**Keywords:** chia seed, long-term dietary intake, musculoskeletal system, bone densitometry analysis, liver, intestine, Sprague-Dawley

## Abstract

Chia seeds (*Salvia hispanica*) provide an unusually high content of α-linolenic acid with several potential health benefits, but few studies have examined the long-term intake of *n*-3 fatty acid-rich plant foods such as chia. In this work, we investigated some of the effects of a diet containing 10% chia seeds versus a conventional isocaloric diet for 10 and 13 months on body measurements, musculoskeletal system, the liver, and the intestines of 20 male Sprague-Dawley rats assigned into two groups. The *n*-6/*n*-3 ratios for the control and chia diets were 7.46 and 1.07, respectively. For the first 10 months of the diet, the body parameters and weights were similar, but at 13 months, the bone mineral content (BMC) of the chia-fed rats was significantly higher than that of the controls whether in total or proximal areas of the left tibia. Also, significant positive correlations were found between the age of the chia group and the bone mineral density, BMC, weight of the musculoskeletal system, final body weight, and skin weight. Liver and intestinal examinations showed improved morphology associated with lower lipid deposit in hepatocytes and increased intestinal muscle layers and crypt size in the chia group. This study provides new data suggesting the potential benefits associated with the long-term intake of chia seeds.

## 1. Introduction

The association of certain foods and dietary habits with several chronic health conditions, including osteoporosis, cardiovascular diseases, diabetes, and some types of cancers, has boosted the search for novel and healthier food options [1,2,3]. Among a diversity of plant foods with high nutritional interest, the chia seed (*Salvia hispanica*) has recently been highlighted for its chemical composition and potential nutritional value [4]. The chia seed contains important quantities of protein, minerals, fiber, polyphenols, and polyunsaturated fatty acids (PUFAs) and is currently known as one of the best plant sources of the omega-3 (*n*-3) fatty acid, α-linolenic acid (ALA) [5,6,7,8,9]. In the face of recent evidence showing the health benefits associated with the intake of foods high in omega-3 [10], the chia seed constitutes an important plant source of *n*-3 PUFA to be explored in different research models for human health and disease prevention.

The musculoskeletal system is made up of muscles, tendons, ligaments, bones, joints, and associated tissues. To achieve its functions, the musculoskeletal system is composed of dynamically active cells and an extracellular matrix [11] sensitive to numerous stimuli and factors that ultimately promote or impair musculoskeletal health [12,13]. Particularly, in the skeleton, for example, some known exogenous factors influencing bone health are associated with the lifestyle category (dietary habits, physical activity/sedentarism, tobacco smoking, sunlight exposure, or vitamin D supplementation) [14,15,16,17], usage of certain medicines [18], and exposure to environmental factors [19]. In addition, some diseases not initially associated with the musculoskeletal system, such as diabetes, are now implicated with increased bone frailty and fracture risk [20]. The mechanisms by which these conditions affect the skeletal system are not yet clearly understood, but dysregulated or unbalanced inflammatory processes seem to be one of the candidates associated with bone loss [21,22]. Some in vitro studies from a researcher of our group have previously shown that fish-purified docosahexaenoic acid (DHA, *n*-3 PUFA) may reduce cell death in fibroblasts and in neurons exposed to oxidative stress triggered by inflammatory stimulus [23,24]. Furthermore, a 2017 clinical trial has shown that the intake of *n*-3 γ-linolenic acid was beneficial for attenuating joint lesions and the disease activity score in patients diagnosed with rheumatoid arthritis [25].

Dietary habits are among the main modifiable elements of the lifestyle. The knowledge of certain ingredients as nutraceuticals and the concept of functional foods emphasize the role of diet quality for health promotion and disease prevention [8,26]. The systematic search for foods with high nutritional density has grown significantly alongside the emergent crisis of chronic diseases worldwide [27]. The growth of the world population, together with aging and unhealthy lifestyle habits, all predispose the increase of chronic conditions, including musculoskeletal conditions [28]. For instance, the importance of *n*-3 PUFA for the musculoskeletal health has been recently highlighted. A growing body of studies suggests that PUFAs may affect bone metabolism [29,30,31]. A 2017 scoping review of several micronutrients for improving health status among older people identified *n-3* fatty acids as selected micronutrients that might effectively improve bone, skeletal muscle, and cognitive function [32]. This suggests that PUFA-rich foods may support musculoskeletal health and attenuate age-related changes in this system. According to the Global Burden of Disease Study 2016, musculoskeletal conditions are among the leading causes of years lived with disability [33]. The prevalence of some musculoskeletal disorders, such as osteoporosis and osteoarthritis, increases with age and is associated with poor health, multimorbidity, polypharmacy, and reduced quality of life [34,35,36,37,38]. Therefore, there is a need for further studies in animals and humans addressing the potential use of foods with respect to the promotion of musculoskeletal health, particularly research considering the outcomes of the consumption of foods with high nutritional values.

Currently, there are few studies that have examined the long-term effect of foods, including of *n*-3 PUFA-rich plant foods, such as the chia seed [39,40,41]. In this work, we investigated some of the effects associated with the long-term intake of chia seeds, namely body weight and composition and the musculoskeletal system during the growing period and adulthood of male Sprague Dawley rats. Based on the findings of recent studies with ALA-rich chia seeds [42,43], we also hypothesized that some parameters associated with the liver and intestinal morphology of these rats would be favorably impacted after completing 10 and 13 months of diet with *S. hispanica* seeds. The data presented in this study address initial questions about the long-term intake of *n*-3 ALA-rich chia seed that may be further explored in nutritional investigations with different approaches focused on examining musculoskeletal health and associated chronic conditions.

## 2. Materials and Methods

### 2.1. Compliance with Ethical Standards

This study was conducted according to the standards of the National Institute of Health Guide for the Care and Use of Laboratory Animals. The research protocol was approved by the Institutional Review Board (registered under the #05.01.2012) and the Committee for the Care and Use of Laboratory Animals (resolution 1 Bis. 07/2013) of the School of Medicine and Health Sciences at Adventist University of River Plate, Argentina. This committee is affiliated with the National Register of Health Research (registered under the #000237) of the Ministry of Health, Argentina.

### 2.2. Dietary Ingredients and Animals

Organic black chia seeds (*Salvia hispanica* L.) were obtained from a national producer in Salta province, northern Argentina. Raw, degummed soybean oil was acquired from Ricedal Alimentos S.A. (Santa Fe, Argentina). Lactic acid casein 90 mesh (95% protein concentration) was obtained from R.S. Albert & Cia S.A. (Buenos Aires, Argentina). Cornstarch, dextrinized corn starch, dextrose monohydrate, and fibers (neutral detergent fibers, concentration greater than 60%, and degradability superior to 80%), were obtained from Corn Products International (Buenos Aires, Argentina). Multivitamins and mineral complexes were manufactured by Vitafor Laboratories (Santa Fe, Argentina). l-cystine was kindly provided by Ningbo Zhenhai Haide Biochem Co., Ltd. (Buenos Aires, Argentina). Table 1 shows the basic composition of the diets.

A total of 20 Sprague-Dawley (Charles River, Raleigh, NC, USA) male rats, 21–23 days old (weight 42–45 g) were obtained from the vivarium of the School of Veterinary Sciences of the University of Buenos Aires. The rats were allowed free water access and food ad libitum and housed in proper animal facilities in the university center under standard temperature conditions of 21–23 °C, humidity of 60–70%, and cycles of 12 h light/dark (8:00 a.m. to 20:00 p.m.). During the first two weeks of acclimatization, the animals were fed a regular, certified rodent diet (GEPSA, Pilar, Argentina). After this period, rats were randomly assigned into two groups of 10 animals per group (the control group vs the chia group). The experimental diets offered to the animals in the control and the chia groups were semisynthetic, isocaloric, and isonitrogenated in relation to each other, and prepared according to the standards of the American Institute of Nutrition-93 (AIN-93M) diet. The experimental diets were given to the animals between the days 35–37 of age (weight 125–130 g) and continued until the end of each assay after approximately 10 and 13 months. Five animals from the chia group and five from the control group were sacrificed after completing 303 days (~10 months) of feeding from the experimental diets, and an equal number of rats were studied after 402 days (~13 months).

Table 2 presents the basic ingredients as sources of energy and the different fatty acid compositions of diets. Chia seeds represented 10% of the chia diet, and during diet preparation, the main seed content (lipids, protein, and minerals) was considered to match the basic composition of the control diet. The control diet contained, per each hundred grams, 15.1 g of proteins, 64.8 g of carbohydrates, and 6.47 g of lipids, while the chia diet contained, 15.4 g of proteins, 62.1 g of carbohydrates, and 6.54 g of lipids, which accounted for 17%, 68%, and 15% of the total calories for both diets, respectively. For the control diet, the lipid composition was mostly soybean oil (95.7% soybean oil, 3.6% casein, and 0.7% cornstarch). In the chia diet, 47.4% of the lipids were derived from soybean oil, 48.6% from chia seeds, and the remaining from casein (3.3%) and cornstarch (0.7%). The ratio of PUFAs supplied by each diet differed due to the chia seeds on the chia diet. The *n*-6/*n*-3 ratio for the control and the chia diets were 7.46 and 1.07, respectively. The chia diet had about 17% more PUFA than the control diet (40.61 g/kg vs. 34.62 g/kg). The supplementation of calcium (calcium carbonate 40% Ca, calcium sulfate dihydrate 23% Ca; with 0.53 g of calcium for the control diet and 0.46 g of calcium for the chia diet to 100g of diet), phosphorus (monopotassium phosphate 23% P, anhydrous disodium phosphate 22% P; with 112.77 ± 2.25 mg of P to 100 g in both diets), and vitamin D (142.86 IU per 100 g in both diets) were offered. In addition, calcium from other ingredients, such as cornstarch and casein, were also counted. In the chia diet, 11.65% of the total calcium was derived from chia seeds. The estimated ratio of calcium of both diets equals one. Choline bitartrate was used at 3.57 g/kg of diet, which gives choline as the free base at 1.43 g/kg of diet. The AIN-93M diet was used as a reference for diet preparation, considering that sexual maturity is reached around 6 weeks of age or between days 40–60.

### 2.3. Preparation of the Food Pellets

Fresh diets were prepared once a week and stored in sealed plastic containers at 4 °C. To achieve optimal mixture homogeneity of the ingredients, a 2 mm particle size was obtained using a sieve analyzer (Zonytest, Buenos Aires, Argentina). The dry ingredients were placed in a bowl, without the vitamins and minerals, and slowly mixed for a few minutes to reduce the dust production. The oil was then gradually added by slow mixing the diet for an additional 8–10 min. The complex of vitamins and minerals was premixed into dextrose powder before being added to the final ingredients under reduced light conditions. The fibers were added just before the process of agglutination in order to create the final product. The last step in the preparation of the diet consisted of the production of the food pellets by gradually drying at a maximum of 90 °C. Figure S1 shows that the final product similar is to commercial rodent pellets. The moisture content of the food pellets (4–7%) was determined by thermogravimetric method with a halogen moisture analyzer (Mettler-Toledo HB43, Columbus, OH, USA).

### 2.4. Body Weight and Composition

Body weight and diet consumption was measured daily throughout the study using an Ohaus portable balance (TA 3001) with a 0.1 g precision. The water intake was also daily assessed by deducting the initial from the final volume of water in the glass containers. At the end of the study, after 12 h of fasting, the rats were sedated, anesthetized with ketamine (80 mg/kg) and xilazine (10 mg/kg), and sacrificed. The body composition was evaluated based on the method described by Cossio-Bolaños [44]. Briefly, three body compartments were assessed, the residual weight (RW), the external fat and skin weight (EFSW), and the fat-free weight (FFW). The RW contained body fluids, visceras, and the internal fat. The EFSW included the total weight of skin and subcutaneous fat tissues and the FFW the musculoskeletal system. The RW was obtained by subtracting, from the total weight, the EFSW and the FFW.

### 2.5. Chemical Analysis of Chia Seeds

The fatty acid composition of the chia seed was determined by gas chromatography with a Shimadzu GC 2014 (Kyoto, Japan) chromatograph equipped with a flame ionization detector. Analyses were carried out with a capillary column CP Sil 88 (100 m, 0.25 μm film thicknesses), and the oven temperature was programmed with an initial temperature of 160 °C with increases of 0.5 °C/min up to 200 °C. The composition of protein, lipids, total dietary fiber, ash, and moisture was determined according to the methodology proposed by AOAC [45].

### 2.6. Bone Assessment

At the completion of each experimental time point, the musculoskeletal system was obtained by dissection and kept frozen at −20°C in sealed plastic bags until the bone mineral content (BMC) and bone mineral density (BMD) of the total skeleton and the total and proximal areas of the left tibia [46] were determined with dual-energy X-ray absorptiometry (DXA, GE Lunar iDXA, GE Healthcare, Buckinghamshire, UK). All rats received a full body scan with software designed for small animals (Lunar DPX-Alpha, Lunar Corp., Madison, WI, USA).

### 2.7. Liver and Intestine Morphology

Histological sections of fragments of the liver and small intestine (10 cm from Treitz angle) of 3 μm thickness were achieved with a microtome (Leica RM2235, Germany) and were stained by toluidine blue and hematoxylin and eosin methods. The slides were examined under the regular light mode of a Carls Zeiss fluorescence microscope (Carl Zeiss Scope.A1) (Thornwood, NY, USA). The ImageJ software (Java, Wayen Rasband, US National Institutes of Health, Bethesda, MD, USA) was used with a grid containing 104 points. The points for the nucleus (~80 nuclei per field), cytoplasm, lipid vesicles and hepatocytes were counted in four different fields per animal [43]. To measure the crypt depth and thickness of the circular and longitudinal muscle layers of the intestine [43], four different fields per animal of histological sections were counted and averages of the thicker and the thinner areas were obtained using the Image-Pro^®^ Premier software version 9.3 (Media Cybernetics, Rockville, MD, USA).

### 2.8. Statistical Analysis

Considering the sample size, to have 80% power to detect differences with a *p* < 0.05 we needed only four animals per group. To account for possible technical problems, we included 5 animals, so we had 90% power to detect a 2% difference between the evaluated parameters from the chia and control groups. The data presented as mean and SD (±) was compared with a Student’s *t*-test and Mann–Whitney U test according to the distribution of the variables. The Spearman correlation was used to analyze the correlation between age and the variables related to weight, body composition, BMC, and BMD. All the statistical analyses were performed using SPSS Inc. (Chicago, IL, USA) version 18.0. The significance level was *p* < 0.05.

## 3. Results

For the first 10 months, the growth and increase in body weight was similar for both groups of animals. Between 10 and 13 months of the experiment, the rats fed with chia seeds, in contrast with the control group, were still growing and changes of weight were seen in different body compartments, including the musculoskeletal system, skin, and visceral weight (Table 3). Bone densitometry analysis confirmed that the rats of the chia group accumulated significantly more minerals in the period of 10–13 months than did the rats of the control group (Table 4).

Dual X-ray body densitometry scans were completed in all animals of each group after 10 and 13 months of receiving the experimental diets, and the bone densitometry analyses of the rats are shown in Table 4. At the first time point, both groups presented similar bone structures indicated by the BMC and BMD determinations of the total body skeleton and the total and proximal areas of the left tibia, used as an optimal area for bone measurement in adult and aged rats [46]. Nevertheless, at the second time point of the study at 13 months, the rats fed with chia seeds showed a significantly higher BMC than did the control rats whether in total or proximal areas of the left tibia (Table 4). Our study also found significant positive correlations linking the aging of the chia group, from 10 to 13 months, with BMD (*p* = 0.005, σ = 0.809), BMC (*p* = 0.010, σ = 0.763) of the total left tibia, and total weight of the musculoskeletal system of the rats (*p* = 0.019, σ = 0.720). Other significant positive correlations of the chia group in this same period of time included the final body weight (*p* = 0.034, σ = 0.671) and the skin weight (*p* = 0.034, σ = 0.671). The rats of the control group did not show significant correlations in bone content and bone density with age. No other significant correlations were found in both groups of rats in the period of time ranging from 10 to 13 months.

**Figure 1:**
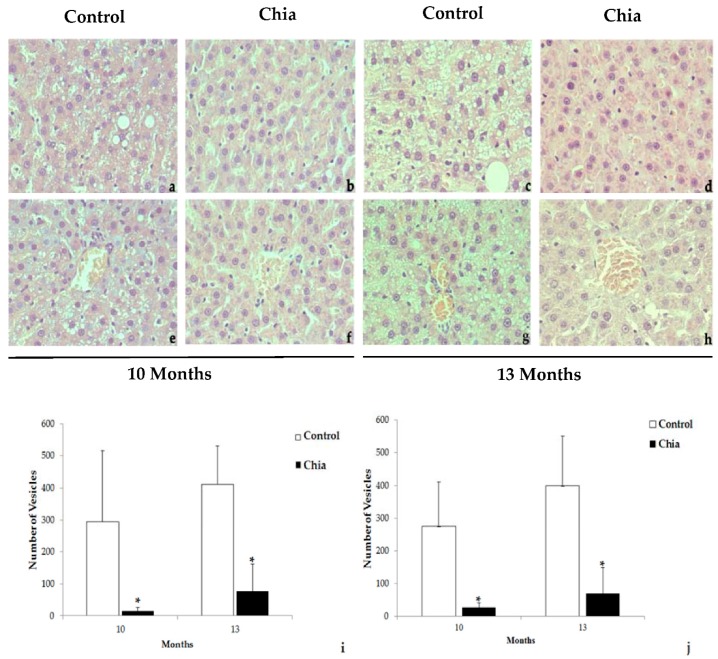
Long-term dietary intake of chia seeds reduces hepatocyte vesicular cytoplasmic structures. Photomicrographs of representative histological sections of the liver, the control group at 10 months (**a**,**e**); the chia group at 10 months (**b**,**f**); the control group at 13 months (**c**,**g**); the chia group at 13 months (**d**,**h**). Microscopic quantification of the hepatocyte vesicular cytoplasmic structures parenchymatic zone at 10 months (*p* = 0.008) and at 13 months (*p* = 0.001) (**i**); central vein zone at 10 months (*p* = 0.008) and at 13 months (*p* = 0.003) (**j**). * *p* < 0.05.

The histological assessment of the liver sections of both groups in our study showed hepatocytes inside the liver lobules and other structures regularly distributed throughout the organ. A significant number of hepatocytes of the control group presented lipid microvesicular structures in the cytoplasm after 10 months of the diet. The number and size of the microvesicles increased in the rats of the control group at 13 months in contrast with the animals fed with chia seeds, which showed significantly fewer vesicles in both time points (Figure 1).

**Figure 2:**
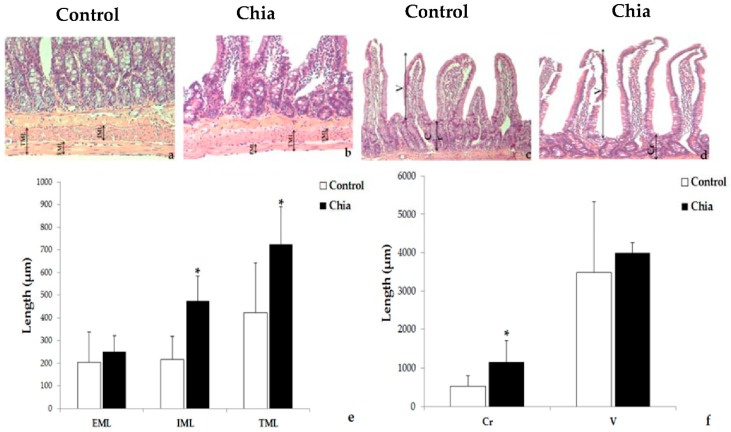
Long-term dietary intake of chia seeds improves intestinal morphology. Photomicrographs of representative histological sections of the small intestinal, showing muscular layers (**a**,**b**); crypt and villus (**c**,**d**). Microscopic quantification of the intestinal muscles’ width, external muscle layer (EML), internal muscle layer IML (*p* = 0.005), total muscle layer TML (*p* = 0.03), (**e**), and crypt Cr (*p* = 0.04), and villus (V) size (**f**). * *p* < 0.05.

The morphology of the intestinal muscle layers, crypt, and villus of a segment of the small intestine is shown in Figure 2. The animals fed with chia seeds after 13 months show, on average, a significantly higher circular internal muscular layer thickness compared with the animals of the control group. The width of the longitudinal external muscular layer was similar in both groups, but the thickness of the two layers together was higher in the rats fed with chia seeds than in the controls. In addition, the average crypt depth of the intestine was considerably larger in the rats treated with chia seeds compared with the animals of the control group, but this was not the case for the size of the villus (Figure 2).

## 4. Discussion

This study explored the effects of the long-term administration of chia seeds, a promising functional plant food with therapeutic perspectives [4,5,6,7,8,9], compared with a conventional diet in an experimental model of Sprague-Dawley rats. Male Sprague-Dawley rats fed a conventional diet tend to increase their bodyweight and reach a plateau of growing at around 12 months of life [47]. However, it is also known that food ingredients and dietary habits play important roles in body metabolism, affecting growth rate, development, cell differentiation, and bodyweight [48,49]. In the study of Poudyal et al. (2012), Wistar rats fed with diets of 5% chia seeds, either included in a regular chow diet or in a high-fat, high-carbohydrate diet, were protected against cardiac and hepatic injuries triggered by an obesogenic diet [41]. Interestingly, in this same study, the intake of chia seeds by the Wistar rats was associated with higher bodyweight and more food consumption than in their counterparts, despite the aforementioned organ protection. These findings are similar to the results of our study, whereby animals fed with chia seeds presented higher intake of food, increased bodyweight, and hepatic protection.

The diet with chia seeds in our study was isocaloric compared with the control diet; nonetheless, the animals of the chia group presented higher food intake. This may be associated with several factors. Some dietary factors, such as food odor and palatability, might have contributed to the amount of food consumed and the bodyweight of the chia group [50,51]. Moreover, the chia seed contains soluble fiber, which may have improved the time of food digestion and the absorption of nutrients by intestinal cells. The physical and chemical characteristics of soluble fiber may alter the absorption of food nutrients, such as fermentation, bulking and binding ability, viscosity and gel formation, water-holding capacity, and solubility [52]. Another aspect particularly associated with the chia diet that might have interfered with the different body weight phenotypes observed in the rats of our study is the potential of the chia seed, as a plant food, to regulate the intestinal microbiome. An increasing number of studies are showing that dietary fiber from plant foods impact gut microbial ecology, host physiology, and health [53,54]. A 2018 study demonstrated that the maturation and anatomy of the enteric nervous system of mice was modulated by the gut microbiota with modifications to the intestinal transit [55]. Also, changes in the microbiota seem to affect food’s satiety [56]. In view of these findings and the health implications associated with the intestinal microbiome, there arises the question of whether chia seeds would promote changes in the gut microbiota, a topic that could be explored in future nutritional evaluations with animals and humans consuming chia seeds or other plant food rich in ALA.

In our investigation, the experimental diets differed in the type of lipids found in chia seeds and soybean oil. The analysis of the chia seed used in this study corroborates other research [6,7,8] in which the *n*-3 ALA was found to be the predominant fatty acid present in this seed (Table S1), in contrast with soybean oil that is richer in *n*-6 linoleic acid [57]. The dietary *n*-6/*n*-3 ratio has been a topic of great interest, because recent evidence suggests that a high *n*-6/*n*-3 ratio is associated with higher risk of several diseases [58,59]. The health benefits of *n*-3 ALA have been highlighted in different studies owing to its potential effect for the prevention of cardiovascular diseases, fracture risk, obesity, and other obesity-associated disorders, such as diabetes [4,60,61]. A recent investigation in animals prone to diabetes found that the intake of chia seeds rich in *n-3* ALA was able to reduce blood levels of pro-inflammatory cytokines and ameliorate insulin sensitivity [62]. In another study with Zucker rats, animals presented similar bodyweights but lower lipid accumulation in the liver when treated with 14% walnut oil in contrast with a diet containing 14% lard [63]. High amounts of *n*-6 in the diet has been associated with abdominal obesity and lipid accumulation in the liver, a condition known as non-alcoholic fatty liver disease [64]. The source and type of lipid in a diet affects metabolism through hormonal signaling affecting the renewal of tissues, lipid redistribution, and changing bodyweight and growth [65,66].

Knowledge of the influence of the nutritional intake of different fatty acids on bone health is limited, but growing evidence suggests that the intake of PUFAs affects bone metabolism [10,11]. The availability of *n*-3 PUFAs in an in vitro study with a RAW264.7 osteoclast differentiation model significantly inhibited the receptor-activated nuclear-kappa B ligand (RANKL)-induced osteoclast formation [67]. The mechanisms by which fatty acids reach bone cells are under investigation. Bartelt et al. recently demonstrated that lipoprotein lipase (LPL) found in bone marrow adipocytes of mice is responsible for triglyceride-rich lipoprotein cleavage and deliverance of fatty acids to specialized bone cells [68]. Under physiologic conditions, bone marrow adipocytes contribute to favor a balanced local fatty acid milieu in bone tissues that is determinant for healthy bone remodeling [68]. The results of this study shed light on the recent understanding of the role of the skeleton, more specifically of bone marrow adipocytes as important regulators of energy metabolism associated with osteogenesis [69].

In our study, the long-term intake of *n*-3 ALA-rich chia seeds may have contributed to the increase of weight of the musculoskeletal system and the changes seen with the DXA analysis of the bone structures of the male rats from 10 to 13 months. Also, the positive correlations found in the chia group of our study presenting higher body weight and musculoskeletal weight is a topic of discussion. The conventional association of high body fat mass with bone mass has been challenged by a number of studies [70,71,72], suggesting that additional mechanisms other than body weight would affect skeletal mass. A study of Bartelt et al. showed that different bone parameters in C57Bl/6 mice were not altered by high-fat diet-induced obesity. On the other hand, in this same research the high-fat diet given to other group of mice, lacking apolipoprotein E and low leptin serum levels, exhibited a high-bone mass phenotype and less fat mass [73]. Interestingly, in this study the researchers showed that the absence of apolipoprotein E expression was not only associated with changes in bone mass but also in marrow adiposity, revealing an intricate interplay of bone cells and fat cells [73]. A recent meta-analysis of studies with overweight and obese human populations corroborates the outcomes of a negative relationship between adipose tissue mass and bone mass [74]. In view of the findings of these investigations, it will be important to determine in future studies the underlying mechanisms of body weight and musculoskeletal weight gain associated with systemic energy balance and adiposity after long-term intake of *n*-3 ALA. The differences in weight observed in the animal groups of our study might be associated with the metabolism of energy and nutrients redistribution. Considering that adipocytes participate in the regulation of energy metabolism it is possible that the chia diet rich in *n*-3 ALA stimulated healthy fat cells to orchestrate fatty acids proper storing in adipose tissues, while excess lipids may have accumulated in other tissues [75], such as observed in the hepatocytes of the control group in our study. Another aspect to be addressed in following investigations is the implication of the dietary intake of *n*-3 fatty acid-rich chia seeds on peroxisome proliferator-activated receptor gamma (PPARγ) activation and adipogenesis. The PPARγ is a member of the nuclear receptor superfamily of ligand-dependent transcription factors functioning as a master regulator of adipocyte differentiation and metabolism [76]. A 2018 review suggests that in addition to the known effect of the PPARγ agonist thiazolidinedione (TZD or glitazones) on fat cells, *n*-3 PUFAs may possibly exert a similar influence stimulating differentiation of adipocytes to a healthy phenotype [77]. Cohort studies conducted in normal and osteopenic women [78] and in healthy young men [79] showed a positive association between serum concentration of *n*-3 PUFAs and total BMD. The low *n*-6/*n*-3 ratio found in the diet with chia seeds may be associated with the findings reported in this study. Indeed, low *n*-6/*n*-3 ratios and diets rich in PUFAs have been shown to affect metabolic rates of tissue renewal, modulating some of the processes associated with aging [80,81]. A 2018 review by Collins and colleagues highlights the connection of metabolic syndrome and obesity, usually associated with low *n*-6/*n*-3 dietary intakes, as a common pathway for low-grade chronic inflammation leading to musculoskeletal impairments, including bone loss and joint inflammation [1]. It will be interesting also to explore the findings of our study in ovariectomized female rats considering that male rats show greater overall body growth and do not experience as marked estrous cycle hormonal fluctuations as females [47]. The study of the musculoskeletal system in male rats supports the need to acquire information about musculoskeletal health in men once they also experience gradual lifetime bone impairments and the age-associated increase of bone fracture risk, although not as severe as seen in postmenopausal women who may suffer from osteoporotic fractures around 10 years earlier in life than men [82].

The formation of microvesicular cytoplasmic structures resembling foamy cells is associated with lipid accumulation in the liver [64]. Silva et al. recently reported that chia seeds, whether offered in the form of flour or whole grain, heat treated or unheated, prevented the usual accumulation of cytoplasmic structures of lipid in the hepatocytes of a healthy non-obese model of Wistar rats [43]. In another study, the chia seed counteracted the hepatocyte accumulation of cytoplasmic lipid vesicular structures in a model of diet-induced obese rats [42]. In our study, the histomorphometric analysis of the muscle layers and crypt of the small intestine of rats differed significantly comparing animals fed with chia and control diets. At least in part, these results may be associated with the type of fiber present in chia seeds, as previously discussed, due to the capacity of the soluble fiber of this seed to increase digestive motility [5,6,7,52,55]. A recent study described similar findings, comparing intestine morphology of Wistar rats fed with different dietary intakes of chia seeds for 28 days [43]. Thus, the intake of chia seeds may be beneficial for improving intestinal transit time that has been lately associated with microbiome health [83].

The limitations of the present study refer to the lack of approaches to assess specific biochemical markers of bone resorption and formation throughout different time points of the study and also the use of micro-computed tomography for further bone evaluations in the rats. However, in humans, densitometry analysis is still the most used approach to evaluate the skeleton considering the radiation hazards associated with computed tomography. Other aspects that could be explored are the impact of the long-term intake of chia seeds on the metabolic health and the identification of specific bone cells affected by the chia diet through histomorphometric analysis.

The impact of PUFAs consumption on human health is currently a topic of great interest. Thus, the extrapolation of the findings of our investigation with chia seeds to future human studies merits some considerations. We have used the whole chia seed instead of plant extracts, such as the oil of this seed rich in *n*-3 ALA. This is important considering the lower cost of the seed in some places of the world, the ease of market accessibility, and the fact that long-chain PUFAs in the form of oils may be oxidized more easily [84]. The results of our study also provide information about a plant-based source of *n*-3 PUFA that is of significance to major dietary patterns, including vegetarian and vegan diets. Also, in view of the growing interest in *n*-3 PUFAs for human diets, there is the risk of excessive supplementation, and the recommended dietary allowance of fatty acids should be followed closely [10]. Considering that the suggested intake of chia seeds for healthy adults is near 25 g/day [4], it is unlikely that overconsumption will occur under regular consumption of foods containing this seed, but this may not be the case when consuming high amounts of supplements or foods fortified with *n*-3 PUFAs. While some studies suggest that excessive intake of PUFAs may promote lean tissue in healthy humans [85], other studies claim an adverse effect in subjects with certain health conditions [86]. We have used the whole chia seed similar to several food preparations for humans containing this seed. Likewise, the chia seed of our study was not exposed to more than 90 °C during food preparation to avoid the possible oxidation of the PUFAs. Interestingly, the chia seed ingredients seem to be easily available for intestinal absorption without the need for grinding the seed prior to consumption [87]. Other nutritional applications have been given to this seed, testing the effects of chia flour and mucilage in different diet preparations with positive results [88,89]. In addition to these aspects, there is some evidence from different studies that *n*-3 PUFAs may be beneficial to bone health; however, only a small number of these investigations assessed the effect of *n*-3 PUFAs derived from plant foods. A recent crossover trial comparing the different dietary intakes of *n*-6/*n*-3 PUFA ratios, derived from plant- and marine-based supplements, did not find significant changes in some bone markers in human young adults after an eight-week feeding period. The authors concluded that additional research with *n*-3 PUFAs should be explored for longer periods of time and in older populations [90]. To evaluate this further, it would be important to examine the effects of the dietary intake of chia seeds in future studies with humans for extended periods of time as similarly proposed in our experimental approach. This may be especially important for adults and old persons considering the age-associated musculoskeletal changes and the potential benefit of *n*-3 ALA-rich chia seeds associated in our study with the skeleton.

Finally, this study is documenting for the first time the effect of the long-term administration of chia seeds in male Sprague-Dawley rats. The assessment of certain body parameters shows differences in the bone mineral content and densitometry analysis and the liver and intestinal morphology favoring the group of rats fed with chia seeds in comparison with the control animals receiving a conventional diet. The findings of this study add to the growing body of literature about the effects of *n*-3 PUFAs and some possible implications for the long-term consumption of chia seeds. 

## Figures and Tables

**Table 1:** Basic composition of diets.

Ingredients (g/kg)	Control Diet	Chia Diet
Cornstarch	463.0	428.0
Casein	151.0	139.0
Dextrinized corn starch	141.0	129.0
Whole chia seed	-	100.0
Dextrose monohydrate	86.0	80.0
Soybean oil	60.0	30.0
Fiber	60.0	60.0
Mineral complex	31.5	28.0
Multivitamins	5.1	5.1
l-cystine	1.8	1.8

**Table 2:** Nutritional composition of the experimental diets.

Nutrients (Unit/kg)	Control Diet	Chia Diet
Total energy (kcal)	3678	3663
Energy as protein (%)	17.0	17.0
Energy as carbohydrate (%)	68.0	68.0
Energy as fat (%)	15.0	15.0
Total fat (g)	64.7	65.3
Saturated fats (g)	9.3	7.8
Monounsaturated fats (g)	13.6	8.9
Polyunsaturated fats (g)	34.6	40.6
*n*-6 fatty acids (g)	30.3	21.0
*n*-3 fatty acids (g)	4.0	19.5
*n*-6/*n*-3 ratio *	7.4	1.0
Total carbohydrate (g)	648.4	621.4
Total protein (g)	150.4	154.9
Vitamins, minerals, fiber, choline **	Based on the AIN-93M guidelines	

* The difference in quantity and quality of the unsaturated fatty acids is due to the insertion of chia seeds 100 g/kg. ** Bitartrate choline 3.57 g/kg diet was used and included in the vitamin mix. AIN-93M: American Institute of Nutrition-93.

**Table 3:** Body composition and food intake of the chia and control groups.

Variables (U)	10 Months		13 Months	
	Control	Chia	Control	Chia
Body weight # (g)	745.9 ± 110.9	786.8 ± 74.6	711.0 ± 58.5	918.2 ± 75.4 *
Body weight gain (%)	497.4 ± 157.1	550.3 ± 93.4	473.0 ± 42.2	608.3 ± 51.5 *
Daily food intake (g/day)	25.6 ± 1.4	30.9 ± 1.7 *	26.6 ± 1.2	31.0 ± 0.7 *
Fat free weight (g)	365.6 ± 24.3	365.0 ± 23.1	356.3 ± 20.3	430.6 ± 44.1 *
Fat weight (g)	204.1 ± 43.3	243.2 ± 25.5	203.4 ± 27.2	288.5 ± 37.1 *
Residual weight (g)	176.1 ± 55.2	178.5 ± 34.0	151.2 ± 14.4	199.0 ± 41.1 *
Water intake (mL/day)	61.9 ± 31.7	61.9 ± 21.4	57.3 ± 26.9	57.9 ± 17.8

U = unit; # initial body weight for control group = 127.76 ± 16.45 and chia group = 121.78 ± 9.88; * *p* < 0.05.

**Table 4:** Bone densitometry analysis of chia and control groups.

Variables	10 Months			13 Months		
	Control	Chia	*p* Value for *t*-Test	Control	Chia	*p* Value for *t*-Test
BMD (mg/cm^2^)	0.34 ± 0.01	0.35 ± 0.01	NS	0.36 ± 0.02	0.37 ± 0.02	NS
BMC	8.69 ± 0.94	9.02 ± 1.01	NS	8.77 ± 1.31	10.05 ± 0.93	NS
BMC tibia left total	0.39 ± 0.03	0.36 ± 0.15	NS *	0.42 ± 0.05	0.51 ± 0.05	0.019
BMD tibia left total (mg/cm^2^)	0.33 ± 0.01	0.34 ± 0.01	NS	0.34 ± 0.01	0.38 ± 0.03	0.052
BMC tibia left proximal	0.15 ± 0.01	0.16 ± 0.02	NS	0.14 ± 0.20	0.17 ± 0.01	0.019
BMD tibia left proximal (mg/cm^2^)	0.40 ± 0.02	0.40 ± 0.02	NS	0.40 ± 0.03	0.44 ± 0.06	NS *

BMD = bone mineral density; BMC = bone mineral content; NS = not significant; * *p* value for Mann–Whitney U test.

## Supplementary Materials

The following are available online at http://www.mdpi.com/2072-6643/10/7/922/s1, Table S1: Nutritional composition of the chia seed studied. Figure S1: Experimental diets in form of food pellets.
