# Supplementary material for: Long-Term Dietary Intake of Chia Seed Is Associated with Increased Bone Mineral Content and Improved Hepatic and Intestinal Morphology in Sprague-Dawley Rats
Source: Nutrients. 2018 Jul 19;10(7):922. doi: 10.3390/nu10070922 (PMC6073254; doi:10.3390/nu10070922)
Supplement: Supplementary file 1 [file nutrients-10-00922-s001.pdf]

Nutrients

Long-term dietary intake of chia seed is associated with increased bone mineral content and improved hepatic and intestinal morphology in Sprague-Dawley rats

Evelyn M. Montes Chañi[1,2], Sandaly O.S. Pacheco[1,2], Gustavo A. Martínez[1], Maykon R. Freitas[1], Joaquin G. Ivona[1], Javier A. Ivona[1], Winston J. Craig[1,3] and Fabio J. Pacheco[1,2]*

[1] Center for Health Sciences Research, School of Medicine & Health Sciences, Universidad Adventista del Plata, Libertador San Martín, 25 de Mayo 99, Entre Ríos, Argentina, 3103; evymar80@gmail.com (E.M.M.C.); gustimart3@gmail.com (G.A.M.); maykonrocha.med@gmail.com (M.R.F.); joaquin.ivona@gmail.com (J.G.I.); javier.ivona@gmail.com (J.A.I.)

[2] Institute for Food Science and Nutrition, Universidad Adventista del Plata, Libertador San Martín, 25 de Mayo 99, Entre Ríos, Argentina, 3103; sandaly.oliveira@uap.edu.ar (S.O.S.P.)

[3] Department of Public Health, Nutrition and Wellness, School of Health Professions, Andrews University, Berrien Springs, Michigan 49104, USA; wcraig@andrews.edu (W.J.C.)

*Correspondence: fabio.pacheco@uap.edu.ar (F.J.P.); Tel: +54-343-4918000, ext. 1236; Fax: +54-343-4910300

Table S1. Nutritional composition of the chia seed studied

Compounds	Concentration (g 100 g^{-1})
Moisture	6,6
Ash	4,73
Total lipids	34,54
Palmitic acid (C16:0)	5,46
Estearic acid (C18:0)	2,14
Oleic acid (c9 C18:1)	4,87
Octadecenoic acid (c11 C18:1)	0,58
Linoleic acid (c9, c12 C18:2)	18,63
γ-linolenic acid (c6, c9, c12 C18:3)	0,22
Arachidic acid (C20:0)	0,16
α-linolenic (c9,c12,c15 C18:3)	67,85

Protein	19,09
Total dietary fiber	16,50
Carbohydrates	18,54
Calcium (mg)	1150,00
Phosphorus (mg)	800,00

Figure S1. Experimental diets in form of food pellets*

Chia diet **Control diet**

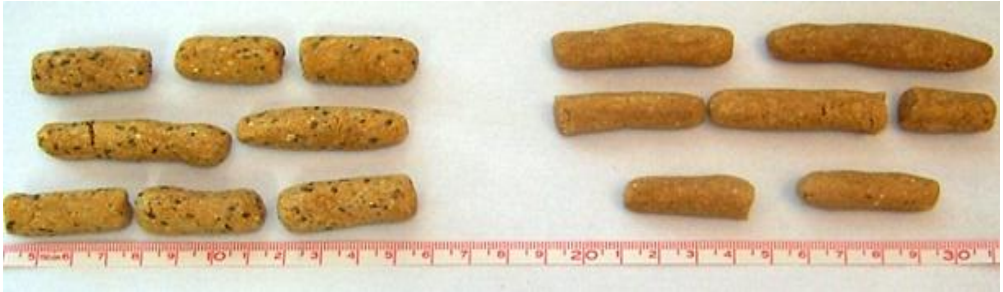

*Unit of the metric ruler in centimeters
